# trioPhaser: using Mendelian inheritance logic to improve genomic phasing of trios

**DOI:** 10.1186/s12859-021-04470-4

**Published:** 2021-11-22

**Authors:** Dustin B. Miller, Stephen R. Piccolo

**Affiliations:** Department of Biology, Brigham Young University, Provo, UT 84602 USA

**Keywords:** Haplotyping, Phasing, Trios, Genomics, Next-generation sequencing

## Abstract

**Background:**

When analyzing DNA sequence data of an individual, knowing which nucleotide was inherited from each parent can be beneficial when trying to identify certain types of DNA variants. Mendelian inheritance logic can be used to accurately phase (haplotype) the majority (67–83%) of an individual's heterozygous nucleotide positions when genotypes are available for both parents (trio). However, when all members of a trio are heterozygous at a position, Mendelian inheritance logic cannot be used to phase. For such positions, a computational phasing algorithm can be used. Existing phasing algorithms use a haplotype reference panel, sequencing reads, and/or parental genotypes to phase an individual; however, they are limited in that they can only phase certain types of variants, require a specific genotype build, require large amounts of storage capacity, and/or require long run times. We created trioPhaser to address these challenges.

**Results:**

trioPhaser uses gVCF files from an individual and their parents as initial input, and then outputs a phased VCF file. Input trio data are first phased using Mendelian inheritance logic. Then, the positions that cannot be phased using inheritance information alone are phased by the *SHAPEIT4* phasing algorithm. Using whole-genome sequencing data of 52 trios, we show that trioPhaser, on average, increases the total number of phased positions by 21.0% and 10.5%, respectively, when compared to the number of positions that *SHAPEIT4* or Mendelian inheritance logic can phase when either is used alone. In addition, we show that the accuracy of the phased calls output by trioPhaser are similar to linked-read and read-backed phasing.

**Conclusion:**

trioPhaser is a containerized software tool that uses both Mendelian inheritance logic and *SHAPEIT4* to phase trios when gVCF files are available. By implementing both phasing methods, more variant positions are phased compared to what either method is able to phase alone.

## Background

When analyzing whole-genome sequence (WGS) data of an individual, the parent of origin for each nucleotide is often unknown [1, 2]. Investigators can identify homozygous-alternate and simple-heterozygous variants, but more complex variants, such as compound-heterozygous variants, are unidentifiable unless the genotype data are phased [3, 4]. Phasing (haplotyping) is a process that helps differentiate between maternally and paternally derived nucleotides [1]. Phase can be estimated with computational methods that rely on haplotype evidence from multiple sources; different software programs may support one or more of the following sources: haplotype reference panel, sequence reads, and/or familial genotypes [1, 5–7]. It has been shown that using trio data (data from each parent and the child) as part of the phasing process can improve the number of accurately phased genotypes [1]. In fact, approximately 67–83% of an individuals’ heterozygous sites can be phased using Mendelian inheritance logic [1, 8].

**Fig. 1:**
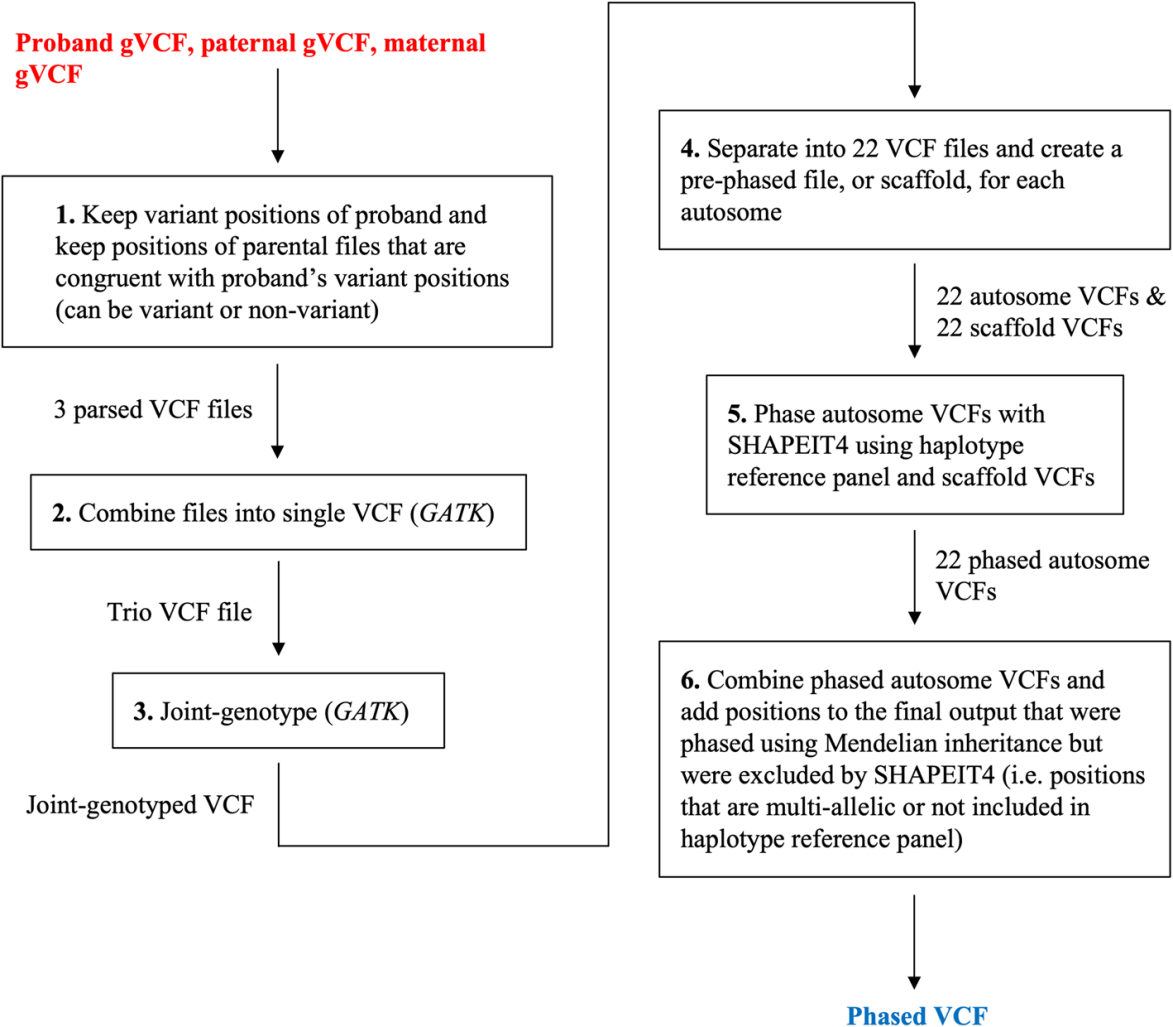
trioPhaser workflow diagram. All steps are conducted within a Docker container. Input files are shown in red and the output file is shown in blue. Steps 1–5 produce temporary output files which are used as input to the subsequent step

Some computational phasing programs, such as *WhatsHap* [6] and *SHAPEIT2* [9] can take parental genotypes into consideration when making haplotype calls for the child. However, these programs have limitations. For example, *WhatsHap* requires BAM and VCF files as input [6]. BAM files from WGS data can be over 100 gigabytes in size, thereby requiring much computational storage when many samples are being analyzed [10]. In addition, *WhatsHap* only outputs phased heterozygous positions, which requires the user to add homozygous alternate positions back into the phased file. *SHAPEIT2* can use a single input type or a combination of input types to estimate phase. Input types supported by *SHAPEIT2* include VCF files, BAM files, a haplotype reference panel, and parental genotypes [9]. However, *SHAPEIT2* does not provide a haplotype reference panel that supports genome build GRCh38. Therefore, VCF files that were generated using GRCh38 require conversion to a previous build through a process that may cause hundreds of thousands of variants to be removed, thereby reducing the total number of variants that are available to be phased [11]. A newer phasing method, *SHAPEIT4*, does not take parental genotype data as direct input into the phasing program, but it does allow pre-phased data to be used as input to increase the accuracy of haplotype calls, and it does support data aligned with GRCh38 [5]. However, *SHAPEIT4* does not provide a means to pre-phase the data, nor does it retain all of the pre-phased data in the final output for variant positions that are not listed in the haplotype reference panel file or that are multi-allelic. Thus, we created trioPhaser, a single-step, containerized application that accepts gVCF files from trio(s) as input, pre-phases the data using Mendelian inheritance logic, and then uses the pre-phased data as input into *SHAPEIT4*, producing a phased VCF file (Fig. 1).

Our method overcomes some of the limitations that current phasing software presents, without creating a completely new phasing algorithm. We are able to use the information that gVCF files provide to pre-phase the data of the child using Mendelian inheritance as a guide. gVCF files are different from VCF files in that both variant and invariant positions are included as part of the file. This proves beneficial as parental genotype information can be compared to a child’s, position for position, in an effort to determine which parent each nucleotide was inherited from. This pre-phase step allows *SHAPEIT4* to use a priori information as part of the phasing process and outputs the data in a conventional format with the paternal allele first, followed by the maternal allele. The phased output file generated by *SHAPEIT4* excludes a priori positions that are not contained in the haplotype reference panel or that are multi-allelic. Therefore, after *SHAPEIT4* phases the data, trioPhaser adds any Mendelian-phased positions to the final output that were excluded.

## Implementation

### Software

trioPhaser consists of a single Python (https://python.org) script (“trio_phaser.py”) that executes within a Docker container [12]. This script provides logic for processing data files and invoking third-party tools including *GATK* [13] (version 4.0.5.1), *bcftools* [14] (version 1.9), and *SHAPEIT4* [5] (version 4.1.3). These tools are available within the container. Therefore, in order to execute trioPhaser, the user needs only to install the Docker engine and download the Docker image. When multiple cores are available, *SHAPEIT4* will phase multiple autosomal chromosomes simultaneously, thereby decreasing run-time.

### Inputs

When a single trio is being phased, trioPhaser has 5 required arguments: (1) gVCF file of the child, (2) gVCF file of the father, (3) gVCF file of the mother, (4) name of the phased output file, and (5) path where haplotype reference files will be saved. Alternatively, when multiple trios need to be phased, trioPhaser has 2 required arguments: (1) a TSV file that contains one trio per line where the first, second, and third columns are file paths to the child, father, and mother gVCF file, respectively, and the fourth column is the path to the output file, and (2) path where haplotype reference files will be saved. There are also 3 optional arguments regardless of whether a single trio or multiple trios are phased: (1) the number of CPU cores to use for processing (default = 2), (2) which genome build the input files were created with (default = GRCh38, GRCh37 is also supported), and (3) the minimum Phred-scaled quality score a variant position is able to have (default = 30).

### Pipeline design

Although trioPhaser executes with a single Python script, there are six steps performed by the script in order to produce a phased VCF file (Fig. 1). For *step 1*, the child’s gVCF file is used to produce a temporary file that contains all variant positions. In addition, the gVCF files for each parent are used to produce temporary VCF files that contain positions that are congruent with the child’s retained variant positions. These positions may be variant or invariant as long as they are found within the newly created file. The purpose of this step is to retain pertinent information and decrease the size of the files. Decreasing file sizes decreases the run-time of subsequent steps.

*Step 2* uses *GATK*’s “CombineGVCFs” tool to combine the temporary VCF files from Step 1 into a single file. Then, *step 3* uses *GATK*’s “GenotypeGVCFs” tool to joint-genotype the calls. Joint-genotyping can help determine if a poorly called variant is a “true” variant by comparing it to the other samples.

Once genotyped, *step 4* separates the file into 22 VCF files and creates a pre-phased file, or scaffold, for each autosome. To create the scaffold files, Mendelian inheritance logic is used. For positions of the child that can be used to determine which variant came from which parent, the haplotypes of these positions are written to a temporary scaffold file. For example, if the mother is homozygotic for the reference allele (A/A), the father is heterozygotic (A/G), and the child is heterozygotic (A/G), then it can be determined that the reference allele (A) was inherited from the mother and the variant allele (G) was inherited from the father. However, when both parents and the child are heterozygotic (A/G), Mendelian inheritance alone cannot be used to determine which nucleotide came from which parent. *SHAPEIT4* uses surrounding pre-phased positions and information from the 1000 Genomes Project [15] haplotype reference panel to phase in such scenarios.

*Step 5* uses the autosome VCF files, the scaffold VCF files, and the haplotype reference panel as input into *SHAPEIT4* to phase each autosome under default parameters. Only positions that are in the haplotype reference panel and that are bi-allelic are included in the phased VCF files output by *SHAPEIT4*. Therefore, *step 6* uses the scaffold files and the *SHAPEIT4*-phased chromosome files to create a single VCF file that includes all Mendelian-phased and *SHAPEIT4*-phased positions for each chromosome. In addition, a separate VCF file is created that only includes phased positions of the child where one of the nucleotides was not identified in either parent. These phased positions may be the result of genotype errors or de novo variants. This file is meant to be informative and allow the user to determine how these positions should be used in downstream analyses.

## Results

### trioPhaser results for 52 trios

Whole-genome sequencing (WGS) data for an Ashkenazim trio and a Han Chinese trio were used to test trioPhaser. Data for these trios were generated by the Genome in a Bottle (GIAB) Consortium [16]. We used GRCh38-aligned BAM files (available through GIAB FTP server) as input into *GATK*’s “HaplotypeCaller” tool to generate gVCF files for each member of each trio. The gVCF files for each trio were then used as input into trioPhaser.

In addition to validating trioPhaser with GIAB data, we ran trioPhaser on 50 trios where the child in each trio had been diagnosed with neuroblastoma. These trios and their associated gVCF files are available through the Gabriella Miller Kids First Data Resource Center [17]. The gVCF files were generated using WGS data.

For the GIAB and 50 neuroblastoma trios, trioPhaser produced a greater number of phased positions than would be provided by using either Mendelian inheritance logic or *SHAPEIT4*, exclusively (Table 1). Mendelian inheritance logic can be used to phase a greater number of positions than *SHAPEIT4* because Mendelian inheritance logic can be applied to multi-allelic positions and does not rely on a haplotype reference panel. However, *SHAPEIT4* provides the added benefit of being able to phase positions where all individuals in a trio are heterozygous for the same nucleotides (given that the positions are bi-allelic and are found within the haplotype reference panel). Overall run-time varies for any given trio based on the number of CPUs available, total number of genotyped variants, and variant Phred-scaled quality threshold. For example, average run-time across the 50 neuroblastoma trios was less than the GIAB trios because at a Phred-scaled quality threshold of 30, there were fewer variants available for phasing than the GIAB trios.

**Table 1:** trioPhaser results for an Ashkenazim trio, Han Chinese trio, and 50 neuroblastoma trios

	Ashkenazim trio	Han Chinese trio	50 neuroblastoma trios
Genotyped variants	5,477,879	5,040,806	5,976,380 sd: 321,208
Variants phased by trioPhaser	4,640,241 (84.7%)	4,490,039 (89.1%)	4,469,479 (75.0%) sd: 171,916
Variants phased exclusively using Mendelian inheritance logic	867,181 (18.7%)	767,992 (17.1%)	721,415 (16.1%) sd: 54,522
Variants phased exclusively by SHAPEIT4	458,361 (9.9%)	417,138 (9.3%)	420,793 (9.4%) sd: 15,414
Run-time (22 CPUs used)	5.8 h	5.4 h	4.4 h

trioPhaser uses Mendelian inheritance in conjunction with *SHAPEIT4*. This hybrid approach produced a greater number of phased positions than was provided using either method exclusively. Results for the 50 neuroblastoma trios were averaged

sd = Standard deviation

### Comparison of trioPhaser to linked-read phasing technology

In addition to BAM files, GIAB provides phased VCF files for each member of the Ashkenazim trio and Han Chinese trio. These data had been phased using linked-read technology (10X Genomics) which is a laboratory-based phasing method that randomly partitions small amounts of DNA to decrease the chance of a single partition having DNA from the same genomic position [1, 18]. This laboratory-based phasing method has been shown to be one of the most accurate [1]. Therefore, we used these trios as our “gold standard” to evaluate how similar the phased results of trioPhaser were to the linked-read technology results.

trioPhaser produced similar phased calls as linked-read phasing technology and is a suitable alternative for phasing trios. For the Ashkenazim trio, trioPhaser and 10X Genomics had 3,999,945 variants that were congruent (those that had the same position, reference allele, and alternate allele). Of the congruent variants, 3,952,525 (98.8%) were phased identically. For the Han Chinese trio, 4,000,682 variants were congruent between the 10X-phased and trioPhaser-phased data; of these variants, 3,900,447 (97.5%) were phased identically.

### Comparison of trioPhaser to WhatsHap

*WhatsHap* is a read-based phasing method that uses sequencing reads to reconstruct haplotypes [6]. This phasing method requires BAM file(s) and a VCF file as inputs. Incorporating sequencing reads as part of the phasing process can increase the overall accuracy of phase results [1]. Therefore, we phased the Ashkenazim and Han Chinese trios using *WhatsHap* to compare the phasing output of read-backed phasing to trioPhaser. Before phasing with *WhatsHap*, we used *GATK*’s “CombineGVCFs” tool to combine the gVCF files we previously created for the Ashkenazim trio and Han Chinese trio, and then joint-genotyped each combined trio using *GATK*’s “GenotypeGVCFs” tool. This joint-genotyped file for each trio and the GRCh38-aligned BAM files for each member of each trio were used as input into *WhatsHap*. The phased VCF output by *WhatsHap* only includes heterozygous positions. Therefore, we only compared heterozygous positions between trioPhaser and *WhatsHap*.

trioPhaser produced similar phased calls on heterozygous positions as *WhatsHap* and is a suitable phasing alternative. For the Ashkenazim trio, trioPhaser and *WhatsHap* had 2,275,546 phased heterozygous variants that were congruent. Of the congruent variants, 2,204,240 (96.9%) were phased identically. 1,959,580 (88.9%) of the congruent positions could be phased using Mendelian inheritance logic and 100% percent of these positions were phased identically between trioPhaser and *WhatsHap*. *WhatsHap* took 26.6 h to phase compared to trioPhaser, which took 5.8 h. For the Han Chinese trio, 2,070,536 phased heterozygous variants were congruent between trioPhaser and *WhatsHap* and of these variants, 1,997,276 (96.5%) were phased identically. 1,777,588 (89.0%) of the congruent positions could be phased using Mendelian inheritance logic, and 100% percent of these positions were phased identically between trioPhaser and *WhatsHap*. *WhatsHap* took 26.6 h to phase compared to trioPhaser, which took 5.4 h.

The source code and a detailed document explaining where data was downloaded from, how it was processed, and how to run trioPhaser is available at https://github.com/dmiller903/trioPhaser.

## Conclusion

trioPhaser is a containerized phasing tool that implements Mendelian inheritance logic and *SHAPEIT4* to phase trios. Using both phasing methods produces a greater overall number of phased variants than would be output when using either method alone. In fact, on average, trioPhaser increased the total number of phased variants by 21.0% and 10.5%, respectively, when compared to what *SHAPEIT4* or Mendelian inheritance logic were able to phase alone. We show that, on average, 98.2% of the congruent 10X-phased haplotype calls, and 96.7% of the congruent *WhatsHap* haplotype calls are the same as trioPhaser. trioPhaser is a suitable phasing alternative for trios when gVCF files are available or can be generated.

## Data Availability

The Genome in a Bottle data underlying this article are available at ftp://ftp-trace.ncbi.nlm.nih.gov/ReferenceSamples/giab/data/AshkenazimTrio/analysis/10XGenomics_ChromiumGenome_LongRanger2.0_06202016/. The Gabriella Miller Kids First data underlying this article cannot be shared publicly due to access restrictions. However, access for these data can be requested through dbGap at https://www.ncbi.nlm.nih.gov/projects/gap/cgi-bin/study.cgi?study_id=phs001436.v1.p1.

## Abbreviations

GIAB: Genome in a bottle
WGS: Whole-genome sequencing
sd: Standard deviation

## Availability and requirements

Project name: *trioPhaser*.

Project home page: https://github.com/dmiller903/trioPhaser.

Project source code: https://github.com/dmiller903/trioPhaser.

Operating system: Linux, Windows, or MacOS.

Programming language: Python3.

Other requirements: *trioPhaser* requires that the Docker engine be installed.

License: MIT.
